# Incidence and Risk Factors for Ventricular Arrhythmias in Patients With Acute Myocardial Infarction Presenting to the Emergency Department

**DOI:** 10.7759/cureus.96527

**Published:** 2025-11-10

**Authors:** Farhan Ali Mazhar, Shafaq Rubab, Asrar Haider, Humaira Sami Ullah, Qazi M Tufail, Usama Tariq, Zain Ramzan

**Affiliations:** 1 Respiratory/Acute Medicine, Russells Hall Hospital, Dudley, GBR; 2 Internal Medicine, Islam Teaching Hospital, Sialkot, PAK; 3 Medicine, Bolan Medical Complex Hospital, Quetta, PAK; 4 Cardiology, Shaikh Zayed Hospital, Lahore, PAK; 5 Medical School, Amna Inayat Medical College, Lahore, PAK

**Keywords:** acute myocardial infarction, hypertension, in-hospital outcomes, patients, risk factors, ventricular arrhythmias, ventricular fibrillation, ventricular tachycardia

## Abstract

Background: Ventricular arrhythmias remain one of the most serious complications of acute myocardial infarction (AMI), often leading to hemodynamic instability and sudden cardiac death.

Objectives: The study aimed to determine the incidence of ventricular arrhythmias in AMI patients presenting to the emergency department, identify associated risk factors, and assess their impact on in-hospital outcomes.

Methodology: This cross-sectional analytical study was conducted at Shaikh Zayed Hospital, Lahore, Pakistan, from March 2024 to September 2024, and included 196 patients diagnosed with AMI. Clinical, demographic, laboratory, and echocardiographic data were recorded. Patients were continuously monitored using ECG during their stay in the emergency department and subsequently throughout hospitalization for up to eight days to detect ventricular arrhythmias, defined as ventricular tachycardia or ventricular fibrillation.

Results: Ventricular arrhythmias were observed in 58 patients (29.6%), including ventricular tachycardia in 19.9% and ventricular fibrillation in 9.7%. Most events (70.7%) occurred within the first 48 hours. Significant predictors included age > 60 years (adjusted odds ratio (AOR) = 1.9), hypertension (AOR = 2.0), diabetes mellitus (AOR = 2.1), anterior wall infarction (AOR = 2.3), Killip class ≥ II (AOR = 2.8), and delayed hospital arrival > 6 hours (AOR = 2.5). Patients with arrhythmias had longer hospital stays (6.2 ± 2.1 vs. 4.8 ± 1.7 days, p = 0.001) and higher in-hospital mortality (20.7% vs. 5.8%, p = 0.001).

Conclusions: Ventricular arrhythmias occurred in nearly one-third of AMI patients, predominantly within the first 48 hours, and were associated with significantly worse in-hospital outcomes. Continuous ECG monitoring during the emergency phase and throughout hospitalization is essential for early detection. The risk factors for ventricular arrhythmias included hypertension, diabetes mellitus, age above 60 years, delayed hospital arrival beyond six hours, low serum potassium, low serum magnesium, and anterior wall infarction.

## Introduction

Acute myocardial infarction (AMI) is one of the most critical cardiovascular emergencies encountered in clinical practice, representing a major cause of morbidity and mortality worldwide. According to the World Health Organization (WHO), ischemic heart disease, including AMI, remains the leading cause of global deaths, accounting for nearly 16% of all deaths annually [1]. Although advances in pharmacological therapy, interventional cardiology, and emergency care have substantially improved patient survival over the past decades, complications such as ventricular arrhythmias continue to pose significant threats to both immediate and long-term outcomes [2]. In fact, ventricular arrhythmias are among the most feared complications of AMI due to their potential to cause hemodynamic collapse, sudden cardiac death, and adverse prognostic implications even in survivors [3]. Ventricular arrhythmias in the setting of AMI are usually classified into two broad categories: early and late. Early arrhythmias typically occur within the first 48 hours of infarction and are largely related to acute myocardial ischemia, electrolyte disturbances, and autonomic imbalance [4]. These arrhythmias may be transient but carry a high risk of clinical deterioration if not promptly managed. In contrast, late ventricular arrhythmias, developing after the initial 48 hours, are often associated with structural myocardial damage, fibrosis, and remodeling, which provide the substrate for re-entrant tachyarrhythmias [5]. Both categories demand close clinical attention, as early arrhythmias threaten immediate survival, while late arrhythmias contribute significantly to long-term mortality and sudden cardiac death. Epidemiological studies have reported varying incidence rates of ventricular arrhythmias among AMI patients, generally ranging from 6% to 20% [6]. These variations reflect differences in study populations, healthcare infrastructure, timing of hospital presentation, and the availability of reperfusion strategies such as thrombolysis and percutaneous coronary intervention (PCI). In high-income countries with well-established acute coronary care pathways, the incidence of life-threatening arrhythmias has declined in recent decades [7]. However, in low- and middle-income countries (LMICs), where delayed presentation and limited access to advanced reperfusion therapies remain prevalent, the burden of arrhythmia-related complications is comparatively higher. This discrepancy underscores the importance of region-specific studies, as risk factors and outcomes may differ significantly based on demographic and healthcare contexts [8].

Multiple clinical and demographic factors have been identified as predictors of ventricular arrhythmias in AMI patients. Age and male gender have consistently been shown to correlate with higher risk, possibly due to greater prevalence of extensive coronary artery disease and structural heart damage in these groups [9]. The site of infarction is another determinant; anterior wall myocardial infarctions are particularly arrhythmogenic due to the larger myocardial territory involved and the consequent disruption of electrical stability. Delayed arrival to hospital and prolonged ischemic time further elevate the risk, as sustained ischemia exacerbates myocardial necrosis and predisposes to electrical instability [10]. Comorbidities such as diabetes mellitus, hypertension, dyslipidemia, and chronic kidney disease also play crucial roles in arrhythmogenesis during AMI. Diabetes, in particular, is associated with autonomic neuropathy, impaired ischemic preconditioning, and a pro-arrhythmic metabolic environment [11]. Similarly, hypertension contributes to left ventricular hypertrophy and increased myocardial oxygen demand, setting the stage for arrhythmia in the presence of acute ischemia. From a clinical standpoint, the occurrence of ventricular arrhythmias in AMI is not merely an acute complication but also a prognostic marker [12]. Early arrhythmias, although sometimes transient, have been associated with increased in-hospital mortality. Late arrhythmias are more ominous, as they often herald the onset of ventricular dysfunction, remodeling, and increased risk of sudden cardiac death [13].

The basic aim of the study was to determine the incidence of ventricular arrhythmias in AMI patients presenting to the emergency department, identify associated risk factors, and assess their impact on in-hospital outcomes.

## Materials and methods

This was a cross-sectional analytical study conducted at Shaikh Zayed Hospital, Lahore, Pakistan, from March 2024 to September 2024. A total of 196 patients diagnosed with AMI were enrolled. Non-probability consecutive sampling was used to recruit eligible participants.

Inclusion criteria

This study included adult patients aged between 30 and 80 years. Both male and female patients were eligible for participation. Only those presenting to the emergency department with a confirmed diagnosis of AMI, established through clinical features, electrocardiographic changes, and elevated cardiac biomarkers, were enrolled.

Exclusion criteria

Patients with a prior history of arrhythmias or conduction abnormalities unrelated to AMI (such as bundle branch block, Wolff-Parkinson-White syndrome, or other pre-existing rhythm disturbances) were excluded. Individuals with congenital or structural heart disease, electrolyte imbalances unrelated to AMI, or incomplete medical records were also excluded.

Data collection

After approval from the institutional ethical review board, informed consent was obtained from patients or their attendants. Detailed clinical evaluation was performed, and demographic data, including age, sex, residence, and comorbidities (diabetes mellitus, hypertension, smoking status, and chronic kidney disease) were recorded. Clinical variables such as type of AMI (ST elevation or non-ST elevation), infarct location, Killip class, and time from symptom onset to hospital arrival were documented. Laboratory parameters (cardiac enzymes, blood glucose, and serum electrolytes) and ECG monitoring were assessed on admission. Patients were monitored continuously in the emergency department for the occurrence of ventricular arrhythmias (ventricular tachycardia or ventricular fibrillation). Patients were monitored for a minimum of 48 hours and up to eight days, depending on clinical stability and discharge timing. 

Statistical analysis

Data were analyzed using IBM SPSS Statistics v26 (IBM Corp., Armonk, NY, USA). Prior to analysis, all continuous variables were evaluated for normality using the Shapiro-Wilk test and for homogeneity of variances using Levene’s test. Normally distributed data were presented as mean ± standard deviation (SD) and compared between groups using the independent samples t-test. Non-normally distributed variables were expressed as median (interquartile range (IQR)) and analyzed using the Mann-Whitney U test. Categorical variables were summarized as frequencies and percentages and compared using the chi-square test or Fisher’s exact test when applicable. To identify independent predictors of ventricular arrhythmias, multivariate binary logistic regression analysis was performed, with results reported as adjusted odds ratios (AORs) and 95% confidence intervals (CIs). A two-tailed p-value ≤ 0.05 was considered statistically significant.

## Results

Data were collected from 196 patients. While 58 (29.6%) developed ventricular arrhythmias, 138 (70.4%) did not. Patients in the arrhythmia group were older, with a mean age of 61.2 ± 10.9 years compared to 57.1 ± 11.9 years in those without arrhythmias. The gender distribution was nearly identical between groups (male: 72.4% vs. 72.5%). Hypertension and diabetes mellitus were more prevalent among patients with arrhythmias (65.5% and 55.2%) compared to those without (50.7% and 39.9%). Similarly, smoking and chronic kidney disease were more frequent in the arrhythmia group (35.9% and 17.2%) than in the non-arrhythmia group (28.9% and 11.6%). Anterior wall myocardial infarction was significantly more common among arrhythmia patients (58.6% vs. 36.2%), and half of them presented with Killip class II or higher, compared to only 26.8% in those without arrhythmias (Table 1).

**Table 1:** Baseline demographic and clinical characteristics of patients (N = 196) Values are expressed as mean ± SD or number (percentage), as appropriate. MI: myocardial infarction; SD: standard deviation

Variable	Ventricular Arrhythmia (n = 58)	No Arrhythmia (n = 138)
Age (years), mean ± SD	61.2 ± 10.9	57.1 ± 11.9
Male, n (%)	42 (72.4)	100 (72.5)
Female, n (%)	16 (27.6)	38 (27.5)
Hypertension, n (%)	38 (65.5)	70 (50.7)
Diabetes mellitus, n (%)	32 (55.2)	55 (39.9)
Smoking, n (%)	21 (35.9)	40 (28.9)
Chronic kidney disease, n (%)	10 (17.2)	16 (11.6)
Anterior wall MI, n (%)	34 (58.6)	50 (36.2)
Killip class ≥ II, n (%)	29 (50.0)	37 (26.8)
Delayed arrival > 6 hours, n (%)	31 (53.4)	43 (31.2)

Overall, ventricular arrhythmias were documented in 58 patients, giving an incidence rate of 29.6%. Ventricular tachycardia 39 (19.9%) was more common than ventricular fibrillation 19 (9.7%). Among these, the majority of arrhythmias, 41 (70.7%), occurred early within 48 hours of infarction, whereas late arrhythmias accounted for 17 (29.3%) (Table 2).

**Table 2:** Incidence and type of ventricular arrhythmias (N = 196)

Category	Subcategory	Frequency (n)	Proportion (%)
Overall arrhythmia status	No arrhythmia	138	70.4
	Ventricular arrhythmia (total)	58	29.6
Type of ventricular arrhythmia	Ventricular tachycardia	39	19.9
	Ventricular fibrillation	19	9.7
Timing of ventricular arrhythmia	Early arrhythmia (≤ 48 hours after infarction)	41	70.7
	Late arrhythmia (> 48 hours after infarction)	17	29.3

Age > 60 years nearly doubled the risk of arrhythmias (AOR = 1.9, p = 0.028). Hypertension (AOR = 2.0, p = 0.031) and diabetes mellitus (AOR = 2.1, p = 0.024) both significantly increased risk. Anterior wall infarction showed more than twofold higher likelihood (AOR = 2.3, p = 0.009). A Killip class ≥ II was the strongest predictor, conferring almost threefold increased risk (AOR = 2.8, p = 0.001) (Table 3).

**Table 3:** Multivariate logistic regression analysis of risk factors for ventricular arrhythmias * p ≤ 0.05; ** p ≤ 0.01. All categorical variables were coded as 1 = presence of characteristic and 0 = absence (reference). No variable exhibited multicollinearity (VIF < 2.0 for all predictors). VIF: variance inflation factor; MI: myocardial infarction; AOR: adjusted odds ratio; CI: confidence interval

Predictor Variable	Reference Group	AOR	95% CI	P-Value	VIF
Age > 60 years	≤ 60 years	1.9	1.1 to 3.6	0.028 *	1.21
Male gender	Female gender	1.3	0.7 to 2.5	0.403	1.17
Hypertension	No hypertension	2.0	1.1 to 3.9	0.031 *	1.34
Diabetes mellitus	No diabetes	2.1	1.1 to 4.0	0.024 *	1.29
Anterior wall MI	Non-anterior MI	2.3	1.2 to 4.2	0.009 **	1.18
Killip class ≥ II	Killip class I	2.8	1.5 to 5.1	0.001 **	1.46
Delayed hospital arrival > 6 hours	≤ 6 hours	2.5	1.3 to 4.6	0.004 **	1.22

Patients with arrhythmias had significantly higher median troponin-I levels (8.1 ng/mL vs. 5.7 ng/mL, p = 0.021), reflecting greater infarct size. Admission random blood glucose was also higher in the arrhythmia group (192 ± 50 mg/dL vs. 171 ± 46 mg/dL, p = 0.018). Electrolyte abnormalities were notable, with lower serum potassium (3.9 vs. 4.2 mEq/L, p = 0.012) and magnesium (1.7 vs. 2.0 mg/dL, p = 0.009) in arrhythmia patients (Table 4).

**Table 4:** Laboratory and echocardiographic parameters of patients (N = 196) * p ≤ 0.05; ** p ≤ 0.01. IQR: interquartile range; CI: confidence interval; SD: standard deviation; df: degrees of freedom; n_1_: arrhythmia group; n_2_: non-arrhythmia group

Variable	Total (N = 196)	Ventricular Arrhythmia (n = 58)	No Arrhythmia (n = 138)	Mean/Median Difference (95% CI)	Test Statistic	P-Value
Troponin-I (ng/mL), median (IQR)	6.4 (3.2-12.8)	8.1 (4.5-14.6)	5.7 (2.9-11.2)	2.4 (95% CI: 0.3 to 4.5)	U = 3058, n₁ = 58, n₂ = 138	0.021 *
Random blood glucose (mg/dL), mean ± SD	178 ± 48	192 ± 50	171 ± 46	21 (95% CI: 4 to 38)	t = 2.39, df = 194	0.018 *
Serum potassium (mEq/L), mean ± SD	4.1 ± 0.6	3.9 ± 0.5	4.2 ± 0.6	-0.3 (95% CI: -0.5 to -0.1)	t = -2.55, df = 194	0.012 *
Serum magnesium (mg/dL), mean ± SD	1.9 ± 0.4	1.7 ± 0.3	2.0 ± 0.4	-0.3 (95% CI: -0.5 to -0.1)	t = -2.64, df = 194	0.009 **
Left ventricular ejection fraction (%), mean ± SD	44.8 ± 8.9	41.2 ± 9.3	46.3 ± 8.5	-5.1 (95% CI: -8.1 to -2.1)	t = -3.31, df = 194	0.001 **

The average length of stay was significantly longer in the arrhythmia group (6.2 ± 2.1 days vs. 4.8 ± 1.7 days, p = 0.001). Heart failure developed in 36.2% of arrhythmia patients compared to 16.7% without arrhythmias (p = 0.004). Cardiogenic shock occurred in 24.1% of patients with arrhythmias versus 8.0% in those without (p = 0.003). Most importantly, in-hospital mortality was more than threefold higher in the arrhythmia group (20.7% vs. 5.8%, p = 0.001) (Table 5).

**Table 5:** In-hospital outcomes of patients with and without ventricular arrhythmias (N = 196) ** p ≤ 0.01. Pearson’s chi-square test was used when expected frequencies ≥ 5; Fisher’s exact test was used when expected frequencies < 5. df: degrees of freedom; SD: standard deviation

Outcome Variable	Ventricular Arrhythmia (n = 58)	No Arrhythmia (n = 138)	Test Statistic	df	P-Value
Length of hospital stay (days), mean ± SD	6.2 ± 2.1	4.8 ± 1.7	t = 3.59	194	0.001 **
Heart failure during admission, n (%)	21 (36.2)	23 (16.7)	χ² = 8.43	1	0.004 **
Cardiogenic shock, n (%)	14 (24.1)	11 (8.0)	χ² = 8.71	1	0.003 **
In-hospital mortality, n (%)	12 (20.7)	8 (5.8)	-	-	0.001 **

## Discussion

This study investigated the incidence and risk factors for ventricular arrhythmias in patients presenting with AMI to the emergency department. Our findings demonstrated that nearly one-third (29.6%) of patients developed ventricular arrhythmias, with ventricular tachycardia being more common than ventricular fibrillation. The majority of arrhythmias occurred within the first 48 hours of infarction, underscoring the importance of early monitoring and intervention during this high-risk period. Although our rate was slightly higher, the incidence rate in this study aligns with previously reported ranges of 6-20% [6]. Our population's higher prevalence of uncontrolled cardiovascular risk factors, limited access to reperfusion therapies, and delays in hospital presentation could be to blame for this. Early ventricular arrhythmias are known to be driven by acute ischemia, metabolic imbalance, and autonomic dysregulation, which likely explains the clustering of events within the first two days of admission [14]. In terms of risk factors, age above 60 years, hypertension, diabetes mellitus, anterior wall infarction, higher Killip class, and delayed hospital arrival emerged as significant predictors of ventricular arrhythmias. These associations are consistent with prior research, which has highlighted the interplay between ischemic burden, comorbidity load, and electrical instability in the myocardium. In this context, hypertension and diabetes are especially significant because they not only accelerate atherosclerosis but also reduce microvascular perfusion, making the myocardium more susceptible to arrhythmogenic triggers [15]. Anterior wall infarction was strongly associated with an increased risk of arrhythmia, reflecting the larger myocardial territory involved and the greater likelihood of conduction system compromise. Similarly, patients in Killip class II or higher had a nearly threefold increased risk of ventricular arrhythmias, emphasizing the prognostic importance of hemodynamic status at presentation. Delayed presentation (> 6 hours) was also a powerful predictor, highlighting the role of ischemic time in determining both infarct size and arrhythmia risk. This finding reinforces the need for public awareness campaigns and streamlined emergency referral pathways to reduce pre-hospital delays in patients with suspected AMI [16]. Our laboratory analysis revealed that patients who developed ventricular arrhythmias had significantly higher troponin levels, lower serum potassium and magnesium levels, and lower left ventricular ejection fraction. These findings are biologically plausible, as larger infarct size and myocardial dysfunction create a substrate for re-entry circuits, while electrolyte imbalances lower the threshold for arrhythmogenesis [17]. In emergency settings, these associations highlight the significance of promptly correcting electrolyte abnormalities and closely monitoring high-troponin patients. Importantly, ventricular arrhythmias were associated with worse in-hospital outcomes, including longer hospital stays, higher rates of heart failure and cardiogenic shock, and significantly greater in-hospital mortality [18]. These results mirror previous evidence showing that arrhythmias in AMI are not merely transient complications but markers of poor prognosis. Early detection and treatment, therefore, remain crucial for reducing adverse outcomes. 

Overall, our findings highlight that ventricular arrhythmias remain a common and clinically significant complication of AMI in our setting. Recognition of high-risk groups such as older patients, those with hypertension, diabetes, anterior infarction, higher Killip class, and delayed presentation can guide physicians in prioritizing continuous ECG monitoring and early aggressive management. Public health interventions to reduce delays in hospital arrival, along with systematic correction of electrolyte imbalances, may further reduce the burden of arrhythmia-related mortality.

Limitations of the study

This study has several limitations. Being a cross-sectional observational analysis, it identifies associations rather than causative relationships between clinical factors and ventricular arrhythmias. Although multivariate logistic regression was performed to adjust for confounders, unmeasured variables may still exist. The duration of ECG monitoring varied from 48 hours to eight days, depending on clinical stability, which may have led to underestimation of late-onset arrhythmias. Multicollinearity was assessed using variance inflation factors (all < 2.0), and no variables were excluded on this basis; however, data on medication use, lipid profiles, and prior cardiac interventions were not available, which could have influenced arrhythmia risk.

## Conclusions

It is concluded that ventricular arrhythmias are a common and clinically significant complication in patients presenting with AMI, with an incidence of nearly one-third in our study population. Ventricular tachycardia was more frequently observed than ventricular fibrillation, and the majority of arrhythmias occurred within the first 48 hours of presentation. Independent predictors included advanced age, hypertension, diabetes mellitus, anterior wall infarction, higher Killip class, and delayed hospital arrival. Patients who developed arrhythmias experienced higher rates of in-hospital complications and mortality. This underscores the importance of early identification and aggressive management of high-risk groups. Thus, early assessment for the risk factors related to ventricular arrhythmias after AMI is significant because it can aid in the implementation of novel risk stratification models and therapeutic methods for decreasing mortality among individuals at high risk.
